# Circulating hypoxia marker carbonic anhydrase IX (CA9) in patients with hepatocellular carcinoma and patients with cirrhosis

**DOI:** 10.1371/journal.pone.0200855

**Published:** 2018-07-16

**Authors:** Fabian Finkelmeier, Özge Canli, Kai-Henrik Peiffer, Dirk Walter, Andrea Tal, Christine Koch, Ursula Pession, Johannes Vermehren, Jörg Trojan, Stefan Zeuzem, Albrecht Piiper, Florian R. Greten, Georgios Grammatikos, Oliver Waidmann

**Affiliations:** 1 Department of Gastroenterology, Hepatology and Endocrinology, University Hospital Frankfurt, Frankfurt/Main, Germany; 2 Georg-Speyer-Haus, Institute for Tumor Biology and Experimental Therapy, Frankfurt, Germany; 3 Department of General and Visceral Surgery, University Hospital Frankfurt, Frankfurt, Germany; University of Navarra School of Medicine and Center for Applied Medical Research (CIMA), SPAIN

## Abstract

**Background and aims:**

Expression of carbonic anhydrase IX (CA9), an enzyme expressed in response to hypoxia, acidosis and oncogenic alterations, is reported to be a prognostic factor in HCC patients. Here we evaluated serum CA9 levels in HCC and cirrhosis patients.

**Methods:**

HCC and cirrhosis patients were prospectively recruited and CA9 levels were determined. CA9 levels were compared to stages of cirrhosis and HCC stages. The association of the CA9 levels and overall survival (OS) was assessed. Furthermore, immunohistochemical CA9 expression in HCC and cirrhosis was evaluated.

**Results:**

215 patients with HCC were included. The median serum CA9 concentration in patients with HCC was 370 pg/ml and significantly higher than in a healthy cohort. Patients with advanced cancer stages (BCLC and ALBI score) had hid significant higher levels of CA9 in the serum. HCC patients with high serum CA9 concentrations (>400 pg/ml) had an increased mortality risk (hazard ratio (HR) 1.690, 95% confidence interval (CI) 1.017–2.809, *P* = 0.043). Serum CA9 concentration in cirrhotic patients did not differ significantly from HCC patients. Higher CA9 levels in cirrhotic patients correlated with portal hypertension and esophageal varices. Patients with ethanol induced cirrhosis had the highest CA9 levels in both cohorts. Levels of CA9 did not correlate with immunohistochemical expression.

**Conclusions:**

We conclude that a high CA9 level is a possible prognostic indicator for a poor outcome in HCC patients. The high CA9 levels are probably mainly associated with portal hypertension. Ductular reactions might be a possible source of serum CA9.

## Introduction

Hepatocellular carcinoma (HCC) is the most common malignant primary liver cancer disease affecting more than half a million patients annually worldwide. Curative HCC treatment is only available in early stages with preserved liver function involving local ablative procedures, surgical resection and liver transplantation[1–3]. The identification of clinical markers correlating with prognosis and stage seems crucial to establish individual treatment plan for HCC patients.

Carbonic anhydrases (CA) are metalloenzymes, which catalyse the rapid reversible interconversion of carbon dioxide to bicarbonate and protons by adding water. The active site contains a zinc ion.[4] There are distinct CA families, the alpha-CAs are found in humans with four more broad subgroups which contain several isoforms (cytosolic, mitochondrial, secreted and membrane associated)[5]. Carbonic anhydrase IX (CA9) is a transmembrane protein with an extracellular catalytic domain regulating the pH in response to hypoxia, acidosis and especially oncogenic alterations. CA9 is a direct transcriptional target of Hif1-alpha[6]. CA9 can produce pericellular bicarbonate ions and after transport to the intracellular compartment thereby neutralize low intracellular pH and is mostly expressed in gastrointestinal and associated soft tissue[7]. Hypoxia is a hallmark of cancer, the reduced oxygen supply leads to glycolysis and thereby an increase of metabolites lowering the intracellular pH[8]. Upregulation of CA9 in tumors is an effective adaptive response to hypoxia and increases the tumor cell survival[9].

Hypoxia in tumours is a known risk factor for poor prognosis and resistance to therapy. Therefore, CA9 has gained interest as a biomarker in malignancies.[10] Increased CA9 serum levels have been associated with outcome and prognosis in prostate cancer[11], renal cell cancer[12–14], gastric cancer[15], breast cancer[16], vulvar cancer[17] and cervical cancer[18]. An evaluation of circulating CA9 in patients with HCC and patients with cirrhosis has not yet been done to our knowledge. The aim of this study was du evaluate CA9 as a serum marker for these patients.

## Patients and methods

### Selection of patients

Between February 2009 and January 2015 patients with confirmed HCC and liver cirrhosis presenting at the Department of Internal Medicine 1 of the Frankfurt University Hospital outpatient clinic were consecutively enrolled into the present prospective cohort study. Parts of this cohort were published in previous biomarker studies[19,20].

Blood samples and patient data used in this study were provided by the University Cancer Center Frankfurt (UCT). Written informed consent was obtained from all patients and the study was approved by the institutional Review Boards of the UCT and the Ethical Committee at the University Hospital Frankfurt (project-number: SGI-04-2017). Serum samples for a healthy control cohort were retrieved from the blood bank of the Red Cross Blood Donation Service. All samples were fully anonymized except for sex and age, all patients gave written informed consent before donation. The study was performed in accordance with the 1975 Declaration of Helsinki and the REMARK guidelines for prospective biomarker studies. The study was approved by the institutional review board of the Frankfurt University Hospital.

HCC was diagnosed according to current guidelines. Inclusion criterion was the diagnosis of HCC[21]. Exclusion criteria were an age below 18 years and a history of cancer other than HCC within the last five years. The BCLC stage determined the treatment of HCC[22]. Patients were assessed by clinical examination, laboratory parameters and the results of ultrasound, CT scans and MRI imaging at the time of inclusion in the study. Blood samples were taken the day patients presented to our clinic and agreed to study inclusion.

The inclusion criteria for the cirrhosis only patients were cirrhosis assessed by liver histopathological examination or pathognomonic results in ultrasound, computed tomography (CT) or magnetic resonance imaging (MRI). Exclusion criteria were patients below 18 years of age or a history of cancer.

### Blood sampling

At the day of study inclusion blood samples were obtained from each subject. In order to remove remaining cells serum tubes were centrifuged at 1500g for 10 min at 4°C, followed by a second centrifugation (2000g for 3 min at 4°C). Thereafter, the serum samples were aliquoted and stored at -80°C. Routine laboratory parameters were measured at the Central Laboratory of the Frankfurt University Hospital.

### ELISA measurements

Samples were blinded for the person who performed measurements and all measurements were determined at the same day from prospectively collected and stored serum samples. CA9 was measured using a commercially available sandwich ELISA (Quantikine Elisa, Human Carbonic Anhydrase IX Immunoassay, DCA900, RND Systems) according to the recommendation of the manufacturer. The minimum detectable level of CA9 is mean 2.28 pg/ml (0.665–4.39 pg/ml). Samples were measured as duplicates. The intra- and inter assay variation were below 20 percent. Markers of cell death, namely M30 and M65 and macrophage activation, namely soluble CD163, were measured as described previously[23–25].

### Immunohistochemical staining and evaluation

Samples were formalin-fixed, paraffin-embedded tissue samples from surgically resected HCCs. Four μm sections were used for histological analysis. For immunohistochemistry after deparaffinization and rehydration of the tissue, antigen retrieval was done by incubating the tissue section in citrate buffer, pH 7.4, in sub boiling temperature for 20 min. Endogenous avidin and biotin were blocked using the avidin-biotin kit (Vector Labs), following the manufacturer’s instructions. Biotin-labeled secondary antibodies (Vectorlabs) were used prior to detection via DAB protocol (Vectorlabs). For counter staining hematoxylin was used. The following primary antibodies was used: anti-CA9 (abcam, ab15086).

Quantification of positive staining area of manually marked tumors was done using eSlide manager version 12.0.1.5027 after image acquisition by the Aperio Scanscope XT (Leica). Pictures for the manuscript at 10x magnification were taken on a Zeiss microscope. The evaluation of the immunostainings was done according to the publication of Kang et al.[26], score 0: no expression, score 1: <5% weakly membranous staining, score 2: 5-<25%, score 3: 25-<50%, score 4: 50-<75%, score 5: >75% moderately to strongly membranous staining.

### Statistical analysis

Patients were followed-up until death or last contact. Continuous variables are shown as means ± standard deviation and categorical variables are reported as frequencies and percentages. Differences in the serum biomarker values between different patient cohorts were determined using the nonparametric Wilcoxon-Mann-Whitney and Kruskal-Wallis tests. For sub-analysis of a statistically significant Kruskal-Wallis test the Bonferroni correction was used. *P* values < 0.05 were considered to be significant. The correlation coefficient r between different parameters was calculated by using the Pearson or Spearman correlation.

Predictors of survival were determined using a univariate Cox regression hazard model. For assessment of independent predictors of survival, a multivariate Cox regression hazard model was used. Survival curves with the estimated hazards were calculated with the Cox regression model. Statistical analyses were performed with SPSS (Version 22.0, IBM, New York, USA). Figures were made by GraphPad Prism version 7.00 for Mac, GraphPad Software, San Diego California USA.

## Results

215 patients (171 males, 79.5% and 44 females, 20.5%) with HCC were prospectively included. Median duration of follow up was 298 with a range of 1–1464 days. 19 (8.8%) patients underwent liver transplantation and 60 (27.9%) patients died within the observation time. The tumor stages of the patients according to the BCLC score and patient characteristics are shown in Table 1.

**Table 1:** Patient characteristics.

Parameter	Patients
**Epidemiology**	
Patients n	215
Gender, m/f (%)	171/44 (79.5/20.5)
Age, median, range	64 (38–86)
**Etiology of liver disease**	
Alcohol abuse, n (%)	73 (34.9%)
Hepatitis C, n (%)	83 (38.6%)
Hepatitis B, n (%)	40 (18.6%)
NASH^1^, n (%)	17 (7.9%)
Cryptogenic, n (%)	15 (7.0%)
Autoimmune, n (%)	1 (0.5%)
**BCLC stage**^2^	
A, n (%)	43 (20.0%)
B, n (%)	89 (41.4%)
C, n (%)	70 (32.6%)
D, n (%)	13 (6.0%)
**Child-Pugh score**	
A, n (%)	133 (61.9%)
B, n (%)	63 (29.3%)
C, n (%)	19 (8.8%)
**Albumin-Bilirubin (ALBI) grade**	
1, n (%)	77 (35.8%)
2, n (%)	105 (48.8%)
3, n (%)	32 (14.9%)
**CLIP**^3^ **Score**	
>2, n (%)	104 (48.4%)
**MELD**^4^**, median, range**	10 (6–36)
**Treatment**	
Resection, n (%)	26 (12.1%)
Local ablation*, n (%)	122 (56.7%)
Sorafenib, n (%)	54 (25.1%)
Liver transplantation, n (%)	19 (8.8%)
**Laboratory results**	
Sodium (mmol/l), median, range	139, 116–148
ALT^5^ (U/l), median, range	56, 7–2131
AST^6^ (U/l), median, range	83, 20–2120
GGT^7^ (U/l), median, range	184, 10–2201
ALP^8^ (U/l), median, range	140, 43–937
Bilirubin (mg/dl), median, range	1.1, 0.2–20.0
Albumin (mg/dl), median, range	3.6, 2–5.0
INR^9^, mean, median, range	1.20, 0.89–3.03
Creatinine (mg/dl), median, range	0.88, 0.11–5.20
CRP^10^ (mg/dl), median, range	0.88, 0.02–34.73
AFP^11^ (ng/ml), median, range	28.6, 1.4–60500

*including Transarterial chemoembolisation (TACE), Radiofrequency ablation (RFA), Laser interstitial thermal therapy (LITT).

Abbreviations:

^1^NASH, non-alcoholic steatohepatitis;

^2^BCLC, Barcelona liver clinic;

^3^CLIP, Cancer of the Liver Italian Program;

^4^MELD, model of end stage liver disease;

^5^ALT, alanine aminotransferase,

^6^AST, aspartate aminotransferase;

^7^GGT, gamma-glutaryl-transferase;

^8^ALP, alkaline phosphatase;

^9^INR, internationalized ratio;

^10^CRP, C-reactive protein;

^11^AFP, alpha-Fetoprotein

### CA9 levels of HCC patients in etiologies and different stages of chronic liver disease

As there is no defined reference level of CA9, CA9 concentrations were determined in the sera from healthy blood donors. 43 patients (28 males, 65.1% and 15 females, 34.9%), with a median age of 57 years (range 37–70) had a median CA9 level of 41 pg/ml (range 5–169 ng/ml). The median serum CA9 concentration in HCC patients was 370 pg/ml (range 10–5080 pg/ml). No differences were found between male and female patients (p = 0.494).

**Fig 1:**
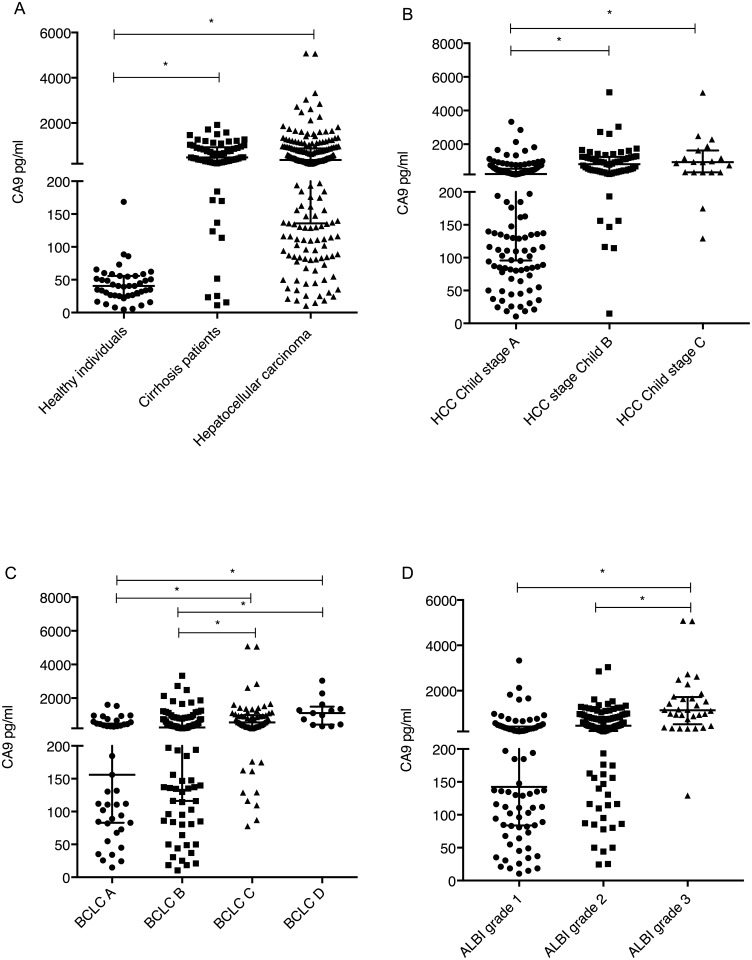
CA9 levels in HCC patients. **A** CA9 levels in healthy patients, patients with cirrhosis only and patients with hepatocellular carcinoma. **B** CA9 levels in different stages of Child Pugh in HCC patients. **C** CA9 levels in different stages of HCC according to BCLC. **D** CA9 levels in different stages of HCC according to ALBI score. (*, P<0.01). Vertical lines indicate the range, the horizontal boundaries of the boxes represent the first and third quartile.

The CA9 levels in HCC patients were significantly higher than those in the healthy cohort (P<0.01) (Fig 1A).

**Fig 2:**
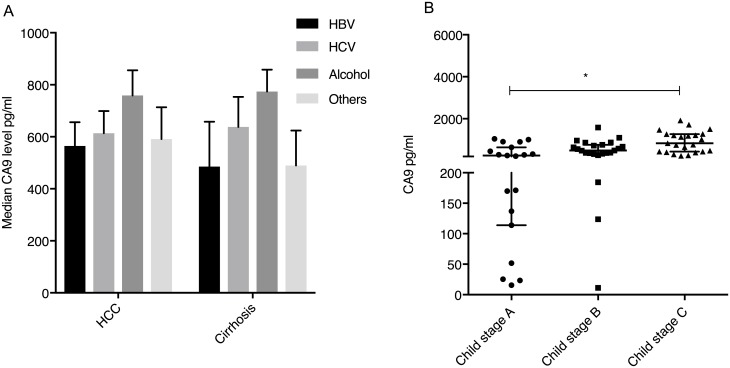
CA9 levels in patients with cirrhosis only and etiology of HCC. **A** Median CA9 levels according to etiology of liver disease in patients with HCC compared to patients with cirrhosis only. (*, P<0.01). **A** CA9 levels in different stages of Child Pugh in cirrhotic patients. Vertical lines indicate the range, the horizontal boundaries of the boxes represent the first and third quartile.

The CA9 levels did not significantly vary in patients with the indicated etiologies of liver disease (*p* = 0.403, *p* = 0.767 and *p* = 0.438, for HBV, HCV infection or NASH, respectively). However, patients with alcoholic liver disease had significantly higher levels of CA9 (median 457 pg/ml, range 19–5066) versus patients with other etiologies (median 331 pg/ml, range 10–5080) (p = 0.028) (Fig 2A).

The CA9 levels in different stages of cirrhosis of HCC patients differed significantly between Child A, B and C patients, with the highest levels found in Child C stage patients (Child A median 228 pg/ml, Child B 810 pg/ml and Child C 930 pg/ml, respectively) (p<0.01) (Fig 1B**)**. There was a significant correlation between the CA9 level and the MELD score (r = 0.414, *p*<0.001).

### CA9 levels within different stages of HCC

The relation of tumor stage and serum CA9 concentrations was investigated. There were significant differences between stages of HCC according to the BCLC staging system, patients with BCLC A or B (median: 245 pg/ml, range 10–3331) had significant lower levels than patients in stage C or D (median: 569 pg/ml, range: 77–5080) (*p*<0.001) (Fig 1C).

Recently a novel staging system for HCC patients based on albumin and bilirubin levels (ALBI-grade)[27] was proposed. The advantage is the minimization of the liver function confounder given in stratifications according to Child-Pugh. The CA9 levels differed significantly between ALBI grade 1, 2 and 3 patients, with the highest levels found in grade 3 patients (Grade 1 median 137 pg/ml, range: 10–3331; Grade 2 median 449 pg/ml, range: 24–3037; and Grade 3 median 1130 pg/ml, range: 129–5080, respectively) (p<0.001) (Fig 1D).

Furthermore, the serum CA9 concentration of HCC patients correlated with systemic inflammatory response, namely CRP levels (r = 0.263, p<0.001) and markers of liver damage, namely GOT levels (r = 0.327, p<0.001) and GGT levels (r = 0.178, p = 0.009). Moreover, there was a modest positive correlation with AFP levels (r = 0.162, p = 0.018).

### CA9 as a risk factor for mortality in HCC patients

As patients with advanced HCC stages had higher serum CA9 concentrations, we hypothesized that CA9 levels might be of prognostic value in HCC patients. According to the median level found in the HCC cohort we chose a cut-off of 400 pg/ml to discriminate patients with high CA9 levels from individuals with lower CA9 levels.

103 patients (47.9%) had CA9 levels higher than 400 pg/ml. Patients with high serum CA9 concentrations had an increased mortality risk (hazard ratio (HR) 1.690, 95% confidence interval (CI) 1.017–2.809, *p* = 0.043).

**Fig 3:**
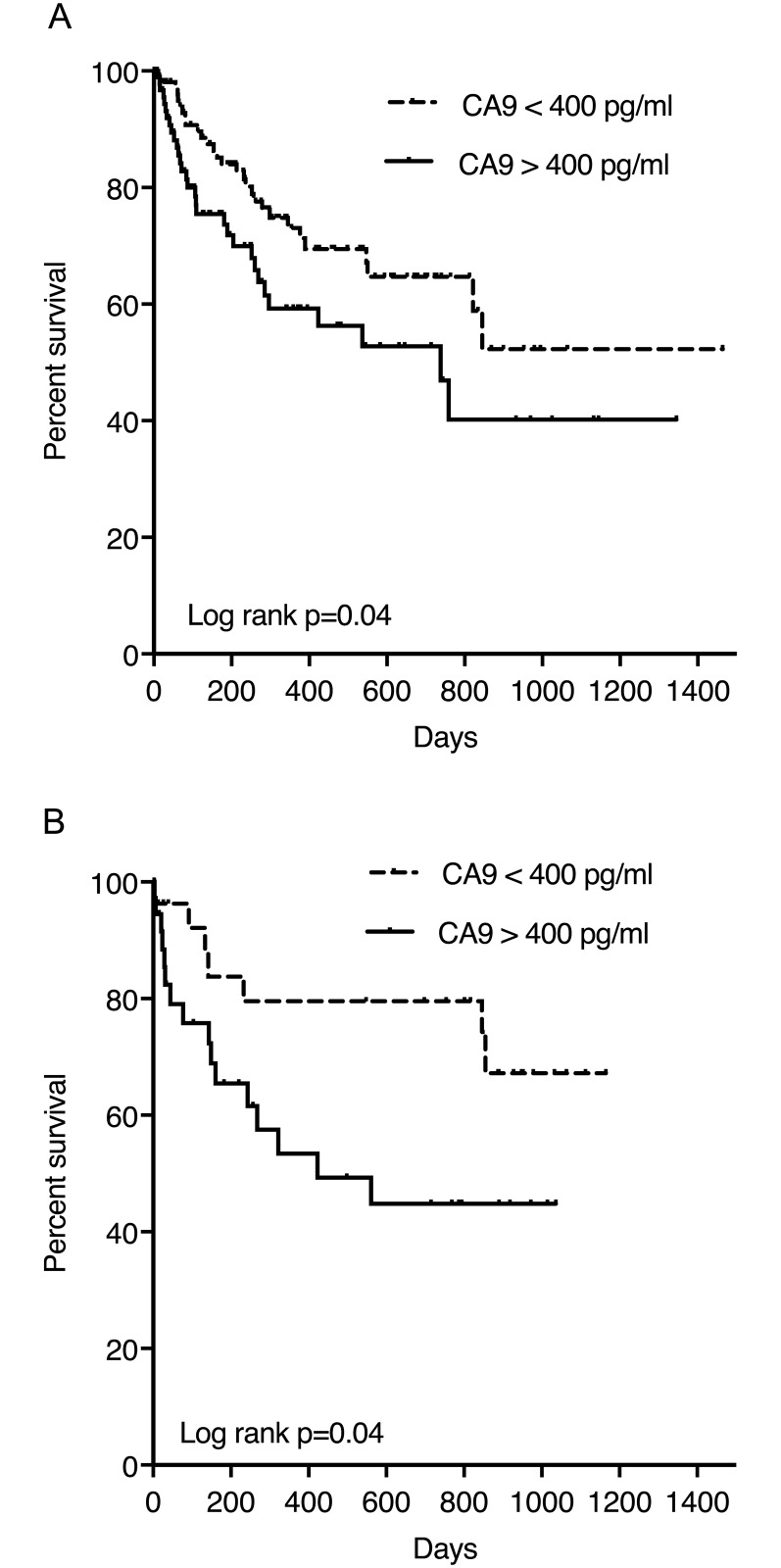
Survival in patients stratified for CA9 levels. **A** Mortality according to CA9 levels in patients with HCC. Patients with high serum CA9 concentrations had an increased mortality risk (hazard ratio (HR) 1.690, 95% confidence interval (CI) 1.017–2.809, *p* = 0.043). **B** Mortality according to CA9 levels in patients with cirrhosis. Patients with high serum CA9 concentrations had an increased mortality risk in this cohort (hazard ratio (HR) 2.458, 95% confidence interval (CI) 1.010–6.114, p = 0.048).

To further analyse the serum CA9 level as prognostic parameter, a multivariate Cox regression model with forward stepwise likelihood ratio was performed. The nominal dichotome variables CA9 (> 400 pg/ml vs. < 400 pg/ml), age (≤ 65 years vs. > 65 years), gender, the BCLC stage and the ALBI grade were included in the model. Only the BCLC stage and ALBI grade levels were independent predictors of survival. **(**Fig 3A, Table 2).

**Table 2:** Univariate and multivariate analyses of parameters associated with overall survival in HCC patients.

	Univariate analysis			Multivariate analysis		
Parameter	HR	95% CI^1^	*P* value	HR^2^	95% CI	*P* value
Male gender	1.045	0.542–2.017	0.895			
Age > 65 years	1.061	0.638–1.765	0.819			
BCLC^3^ stage	1.713	1.197–2.450	**0.003**	1.535	1.079–2.184	**0.017**
Albumin-Bilirubin (ALBI) grade	2.082	1.425–3.041	**<0.001**	1.970	1.326–2.928	**0.001**
CA9^4^ levels > 400 pg/ml	1.690	1.017–2.809	**0.043**			

Abbreviations:

^1^CI, confidence interval;

^2^HR, hazard ration;

^3^BCLC, Barcelona liver clinic;

^4^ CA9, carboanhydrase 9

### CA9 levels in patients with cirrhosis without HCC

As CA9 levels showed a high correlation with stage of cirrhosis and was not an independent factor for survival, we evaluated 65 patients, matched according to stage of cirrhosis, without HCC. The patient characteristics are shown in Table 3.

**Table 3:** Patient characteristics of patients with cirrhosis.

Parameter	Patients
**Epidemiology**	
Patients n	65
Gender, m/f (%)	42/23 (64.6/35.4)
Age, median, range	75 (25–79)
**Etiology of liver disease**	
Alcohol abuse, n (%)	33 (50.8)
Hepatitis C, n (%)	14 (21.5)
Hepatitis B, n (%)	7 (10.8)
NASH^1^, n (%)	1 (1.5)
Cryptogenic, n (%)	6 (9.2)
Autoimmune, n (%)	4 (6.2)
**Child-Pugh score**	
A, n (%)	19 (29.2)
B, n (%)	23 (35.4)
C, n (%)	23 (35.4)
**MELD**^2^**, median, range**	16, 7–40
**Laboratory results**	
Sodium (mmol/l), median, range	139, 128–148
ALT^3^ (U/l), median, range	31, 2–248
AST^4^ (U/l), median, range	52, 18–415
GGT^5^ (U/l), median, range	98, 20–1178
ALP^6^ (U/l), median, range	116, 36–504
Bilirubin (mg/dl), median, range	2.5, 0.2–51.0
Albumin (mg/dl), median, range	3.3, 1.9–5.2
INR^7^, mean, median, range	1.47, 0.85–4.2
Creatinine (mg/dl), median, range	
CRP^8^ (mg/dl), median, range	

Abbreviations:

^1^NASH, non-alcoholic steatohepatitis;

^2^MELD, model of end stage liver disease;

^3^ALT, alanine aminotransferase,

^4^AST, aspartate aminotransferase;

^5^GGT, gamma-glutaryl-transferase;

^6^ALP, alkaline phosphatase;

^7^INR, internationalized ratio;

^8^CRP, C-reactive protein;

The median serum CA9 concentration in cirrhosis patients was 482 pg/ml (range 11–1921). No differences were found between male and female patients (p = 0.869).

CA9 levels in cirrhotic patients were significantly higher than CA9 determined in the healthy cohort (p<0.01), but not different from HCC patients (Fig 1A).

The CA9 levels did not significantly vary in patients with HBV (p = 0.310) or HCV (p = 0.714). Similar to the HCC cohort patients with alcoholic liver disease had significantly higher levels of CA9 (median 750 pg/ml, 16–1921) as compared to patients with other etiologies (median 367 pg/ml, 11–1470) (p = 0.008) (Fig 2A).

The CA9 levels in different stages of cirrhosis differed significantly between Child A, B and C patients, with the highest levels found in Child C stage patients (Child A median 252 pg/ml, Child B 490 pg/ml and Child C 833 pg/ml, respectively) (p<0.01) (Fig 2B). CA9 levels correlated significantly with MELD score (r = 0.483, p<0.001) of the patients.

Soluble CD163 (sCD163) is shed in the blood circulation by activated macrophages and correlates strongly with the hepatic venous pressure gradient (HVPG) and is thereby a good indicator of portal hypertension[24]. A low platelet count is also associated with portal hypertension. As CA9 increased with impaired liver function, we concluded that platelet count as well as sCD163 levels might correlate with CA9 levels. CA9 levels correlated positively with sCD163 (r = 0.481, p<0.001) and negatively with thrombocyte count (r = -0.184, p = 0.01). Patients with diagnosed esophageal varices had significant higher levels of CA9 (median 667 pg/ml, 11–1921) than patients without varices (median 319 pg/ml, 23–1277) (p = 0.03).

Soluble CA9 levels did not correlate with cell death markers in the sera of patients, namely M30 (r = 0.247, p = 0.074) and M65 (r = 0.202, p = 0.106), which were shown to be a prognostic marker of liver cirrhosis[23].

### CA9 as a risk factor for mortality in cirrhosis patients

As patients with advanced cirrhosis stages had higher serum CA9 concentrations, we evaluated the prognostic value of CA9 in these patients with the cut-off of 400 pg/ml according to the median level.

38 patients (58.5%) had CA9 levels higher than 400 pg/ml. Patients with high serum CA9 concentrations had an increased mortality risk in this cohort (hazard ratio (HR) 2.458, 95% confidence interval (CI) 1.010–6.114, p = 0.048) (Fig 3B).

### Immunohistochemistry of CA9 in patients with resected HCC

To evaluate if high levels of soluble CA9 correlate with positive CA9 in tumorous tissue we evaluated 16 patient samples of HCC patients who were resected at our hospital and tissue and serum samples were available. From all patient’s tumors and non-tumorous tissue was stained, all patients had proven cirrhosis.

**Fig 4:**
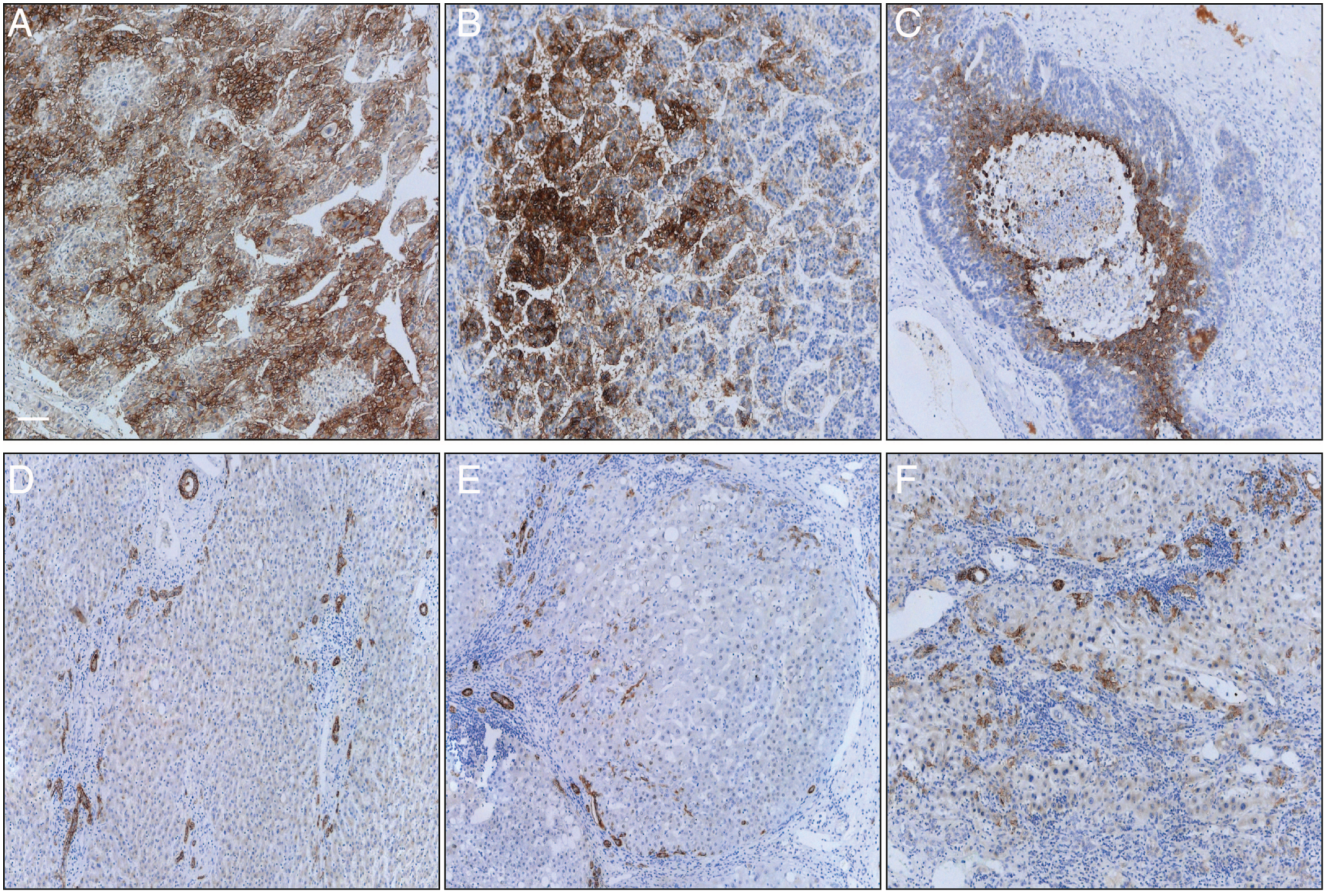
CA9 immunohistochemistry of HCC and cirrhosis samples. Representative pictures (magnification 10x) of CA9 staining. White bar represents 100μm. **A** HCC of a patient with cryptic cirrhosis, CA9 score 4. **B** HCC of a patient with HCV cirrhosis, CA9 score 3. **C** HCC of a patient with ethanol induced cirrhosis and necrotic area, CA9 score 3. **D** Non-tumorous area in cryptic cirrhosis with ductular reactions. **E** Non-tumorous area in NASH cirrhosis with ductular reactions. **F** Non-tumorous area in ethanol induced cirrhosis with ductular reactions. The evaluation of the immunostainings was done according to the publication of Kang et al.[26], score 0: no expression, score 1: <5% weakly membranous staining, score 2: 5-<25%, score 3: 25-<50%, score 4: 50-<75%, score 5: >75% moderately to strongly membranous staining.

9 patients (56.25%) were positive for CA9, two patients (12.5%) had a high staining score of 4 (50–75% of cell membranes moderately to strongly stained), 7 (43.75%) patients had a score of 1, which is counted as negative staining (<5% positive membranes). In this small cohort expression of CA9 did not correlate with the level of CA9 in the serum (0.305, p = 0.286). CA9 staining of cirrhotic tissue showed positive staining of normal bile ducts and an increased number of irregular counterparts, a process known as *ductular reaction*. Furthermore, in cirrhosis single hepatocytes showed positivity for CA9. (Table 4 and Fig 4).

**Table 4:** Patient characteristics and tumor stage of CA9 immunohistochemistry in HCC specimen.

Patient	Sex	Age	Child-Pugh	MELD	Tumorstage	Liver disease	CA9 level pg/ml	IHC Score
1	male	70	B	11	G2, pT1, pN0, V0, L0, R0	Hemochromatosis	543,27	1
2		67	A	11	G2, pT2, pN0, V1, L0, R0	Cryptic	410,23	4
3	male	65	A	9	G2, pT3, pN0, V1, L0, R1.	HCV	323,5	2
4	male	66	A	13	G2, pT1, pN0, V0, L0, R0.	HCV	440	3
5	male	72	A	10	G2, pT3, pN0, V0, L0, R0	Cryptic	87	1
6	male	68	A	17	G2, pT2, pN0, V1, L0, R0.	NASH	80	1
7	male	40	A	14	G3, pT3, pN0, V1, L0, R1	HBV	1253	4
8	male	73	A	10	G2, pT2, pN0, V1, L0, R0	HCV		1
9	male	69	A	16	G2, pT2, pN0, V1, L0, R0	HBV	102	2
10	male	70	A	9	G2, pT2, pN0, L0, V0, R1	Alcohol	845	1
11	male	41	A	7	G2, pT1, pN0, L0, V0, R0	Alcohol	137	1
12	male	52	A	7	G2, pT3b, pN0, L1, V1, R1	Alcohol	30,58	3
13	male	72	A	8	G3, pT2, pN0, L0, V1, R0	HCV	448,59	2
14	female	50	A	8	G2, pT1 pN0, L0, V0, R0	Alcohol	201,77	3
15	male	81	A	7	G2, pT1, pN0, L0, V0, R0	HBV	102,84	1
16	female	72	A	9	G2, pT1, pN0, L0, V0, R0	HCV	110,68	3

## Discussion

CA9 expression is low to undetectable in normal tissue while it is abundant in several tumors[28]. In 2014 Luong-Player et al. evaluated 1125 malignant tumors and normal tissue for immunohistochemical CA9 expression. In their study 28 cases of normal livers were evaluated showing all a strong CA9 expression in the bile ducts, but not in hepatocytes. However, from 34 HCC cases only 15% showed a positive staining. The authors suggested CA9 as a potential marker for the differentiation of cholangiocellular carcinoma (CCA) and hepatocellular carcinoma as 90% of CCA showed a strong CA9 expression[29].

Two groups evaluated the expression of CA9 in resected, early stage HCC. Kang et al. performed CA9 IHC on tissue microarrays of 838 patients and a validation cohort of 225 patients with resected HCC. They found CA9 positivity (>5% staining area) in 15–20% of the patients with a significant correlation with shorter overall survival. However, they found no CA9 expression in non-neoplastic tissue, which is interesting as patients with cirrhosis in our cohort had increased levels of CA9 as well and showed distinct positivity in ductular reactions. Huang *et al*. evaluated 227 unifocal, resected primary HCCs for CA9 expression. 48.5% of all patients showed mainly membranous CA9 expression correlating with younger age, female sex, higher AFP levels, tumor size grading and even survival. In non-tumoral tissue CA9 was almost exclusively expressed in biliary epithelial cells.[30] Lai et al. evaluated CA9 immunohistochemically in patients who underwent TACE therapy. They found a significant upregulation of CA9 in TACE treated nodules (47.5%) compared to non-treated patients (11.8%). Again, CA9 positivity in non-neoplastic parenchyma was only found in bile duct cells.

Taken together HCCs express CA9 only in up to 50% of the cases, especially when necrosis is present. Epithelial cells of the bile duct always express CA9. These findings are comparable to our data on CA9 expression. However, the increased positivity in cirrhosis, probably due to irregular bile ducts, presented here is novel.

Serum CA9 serum levels have not been investigated in HCC patients before. In our study high CA9 levels were of prognostic relevance in HCC and correlated with advanced stage disease, which is in line with previous reports which found high levels of CA9 expression in tissue analyses in patients with advanced HCC. However, patients with cirrhosis and without HCC also showed increased levels of CA9 which were comparable to cirrhotic patients with HCC. Interestingly, immunohistochemical expression of CA9 did not correlate with serum levels of CA9. This raises the main question of the source of serum CA9, which is not clearly understood. The extracellular domain (ECD) of CA9 can be released into the body fluids of (tumor) patients, where it is measured as serum CA9. The release is partially an active metalloprotease-mediated process, that is regulated by different signals and may therefore participate in adaptive changes in the protein composition of tumor cells and of their microenvironment, however, only 10–20% of soluble CA9 is produced by this mechanism [31,32]. Recently, it was shown, that CA9 ECD is increased in response to induction of apoptosis in tumor cells or by treatment with chemotherapeutic drugs presenting cell death as another source of CA9[33]. Ongoing cell death and shedding may explain elevated CA9 levels in tumor patients, however the source in cirrhotic patients remains to be elucidated. In our cirrhosis cohort, there was no correlation of CA9 to markers of cell death (M30, M65), which argues against apoptotic or necrotic cell death as the main source of CA9.

We can only speculate, but a possible role in cell migration and EMT could explain the expression in epithelial biliary cells and especially in ductular reactions. Ductular reactions (DR) are a reactive process of expansion of epithelial cells arising in disease and injury of the liver at the interface of the portal and the parenchymal compartment in human livers. They do not exist in normal livers. The composition of DRs consists of a diversity of cells, the main components are hepatobiliary epithelial cells (the ductular component). DRs are complex networks of hepatobiliary cells with features of proliferative progenitor cells, branching from the Canals of Hering, being a part of the complex self-repair system of the liver[34]. Interestingly DRs correlate with worsening stage of chronic liver disease and oxidative stress seems to play a role, which would fit this hypothesis[35,36]. Suggesting DRs as at least one source of CA9 in non-tumorous but cirrhotic liver tissue remains absolutely speculative, however, this warrants further investigation.

Subgroup analysis yielded significantly higher CA9 levels in both cohorts (HCC and without HCC) in patients with alcoholic liver disease compared to other diseases. CA9 is a very sensitive marker for Hif1-alpha activation due to the unique structure of the CA9 promoter and basically represents the transcriptional activity of HIF1alpha[37]. Ethanol leads to oxidative stress in livers, however, the role of Hif1alpha in alcoholic liver disease (ALD) is still contradictory[38]. Interestingly CA9 levels correlated with markers of portal hypertension (soluble CD163 and platelet count) and is significantly increased in patients with diagnosed esophageal varices supporting further investigations in cirrhotic patients and as a marker for portal hypertension. As CA9 is mainly expressed in the gastrointestinal tract, portal hypertension and associated comorbidities (e.g. hypertensive gastropathy) could be another source of high CA9 levels.

Comparing CA9 levels in cirrhotic patients with and without HCC shows that CA9 showed comparable upregulation in both cohorts. However, higher levels of CA9 correlated with advanced stage HCC and survival. The source of serum CA9, especially in cirrhosis, remains unclear and needs further investigation, especially because of the missing correlation of positive IHC and serum CA9 levels. Ductular reactions in liver cirrhosis may be a source. As CA9 expression is limited to only a few normal tissues, mainly the epithelia of the gastrointestinal tract, an elevated level however, is a potential marker for liver disease. The prognostic value in patients with cirrhosis only should be further evaluated in the future.

## Abbreviations

ALBI: Albumin-Bilirubin grade
ALD: Alcholic liver disease
BCLC: Barcelona Clinic Liver Cancer
CA9: Carboanhydrase 9
CCC: Cholangiocellular carcinoma
CI: Confidence intervall
CLIP: Cancer of the Liver Italian Program
CT: Computed tomography
DR: Ductular reaction
ECD: Extracellular domain
EMT: Epithelial mesenchymal transition
HBV: Hepatitis B virus
HCC: Hepatocellular carcinoma
HCV: Hepatitis C virus
HR: Hazard ratio
IHC: Immunohistochemistry
MELD: Model end stage liver disease
MRI: Magnetic resonance imaging
NASH: Non-alcoholic steatohepatitis
OS: Overall survival
TACE: Transarterial chemoembolisation
